# Serum Heavy Metal Burden and Its Association With Angiographic Severity and Six-Month Outcomes in Coronary Artery Disease: A Prospective Observational Study From North India

**DOI:** 10.7759/cureus.95177

**Published:** 2025-10-22

**Authors:** Ashish Jha, Bhuwan C Tiwari, Aditya C Upadhyay, Sudarshan K Vijay, Naveen Jamwal, Amresh Kumar Singh, Sandeepan Saha

**Affiliations:** 1 Cardiology, Dr. Ram Manohar Lohia Institute of Medical Sciences, Lucknow, IND

**Keywords:** arsenic, cadmium, cardiovascular risk, coronary artery disease, environmental toxins, heavy metals, mercury

## Abstract

Background: Heavy metal exposure is a silent yet significant cardiovascular risk factor, particularly in low- and middle-income countries with evolving environmental and dietary patterns. Metals like cadmium, arsenic, and mercury contribute to atherogenesis through oxidative stress, inflammation, and endothelial dysfunction. However, their association with coronary artery disease (CAD) severity and clinical outcomes remains underexplored in Indian populations. This study evaluated serum levels of six heavy metals and their relation to angiographic complexity and six-month cardiovascular outcomes.

Methods: In this single-center, prospective study, 497 patients undergoing coronary angiography for CAD were enrolled and stratified by the SYNTAX score: SYNTAX score <23 and SYNTAX score ≥23. Serum levels of arsenic, antimony, mercury, lead, nickel, and cadmium were quantified using inductively coupled plasma mass spectrometry (ICP-MS). SYNTAX score, clinical parameters, and six-month outcomes for major adverse cardiovascular events (MACE) were recorded.

Results: Mean SYNTAX scores did not differ significantly across quartiles of any heavy metal (all p > 0.05). Adjusted analysis showed no association between metal levels and high SYNTAX score (adjusted OR = 1.07, 95% CI 0.89-1.30, for cadmium per SD increase). However, subgroup analysis revealed higher cadmium levels in patients with acute coronary syndrome (ACS) compared to stable CAD patients (median = 0.78 (IQR 0.65-0.94) µg/L vs. 0.62 (IQR 0.54-0.75) µg/L; p = 0.006), and elevated arsenic and mercury levels in hypertensive patients (mean difference = +1.21 µg/L (95% CI 0.58-1.84), p = 0.001; +0.34 µg/L (95% CI 0.15-0.53), p = 0.0013). At the six-month follow-up, MACE occurred in 6.6% of patients and were associated with a high SYNTAX score (OR = 4.4 (95% CI 1.7-11.5), p = 0.002), but not with any metal level (all p > 0.1).

Conclusion: Serum heavy metal levels were not associated with angiographic CAD severity. Nevertheless, higher cadmium levels in ACS patients and elevated arsenic/mercury levels in hypertensive patients suggest that metal exposure may influence disease phenotype rather than plaque burden. These exploratory findings warrant validation in larger longitudinal cohorts.

## Introduction

Cardiovascular diseases (CVDs) are the leading cause of death and disability in India, with the CVD death rate of 272 per 100,000 population in India, compared to the global average of 235 per 100,000 [1]. This clearly indicates that India has a significantly higher burden of CVD [2]. Notably, Indians tend to develop CVD nearly a decade earlier than their Western counterparts, a trend that has long concerned clinicians and researchers [3].

While conventional risk factors such as hypertension, diabetes, dyslipidemia, and smoking are well established, they do not fully explain the earlier onset and more severe coronary artery disease (CAD). In a landmark meta-analysis, Nair et al. proposed that unconventional and conditioning risk factors, such as chronic stress, inflammation, and lipoprotein(a), may act synergistically with traditional risks to accelerate atherosclerosis in South Asians [4]. However, despite the increasing control of these known variables, the epidemic of premature CAD persists, suggesting the presence of additional, underexplored modifiable risk factors.

In the context of rapid industrialization and urbanization, environmental toxicants, particularly heavy metals, represent a neglected but biologically plausible class of cardiovascular risk factors. Metals such as cadmium, arsenic, and mercury exert vascular toxicity primarily through oxidative stress, endothelial dysfunction, and inflammation. These metals catalyze the generation of reactive oxygen species (ROS), decrease endothelial nitric oxide synthase (eNOS) activity, promote lipid peroxidation, and upregulate adhesion molecules (vascular cell adhesion molecule-1, or VCAM-1; intercellular adhesion molecule-1, or ICAM-1), culminating in vascular injury, stiffness, and plaque vulnerability [5,6]. Chronic low-level exposure may thus influence not only atherosclerosis progression but also plaque stability and blood pressure regulation.

The selection of the six metals in this study, i.e., arsenic, cadmium, mercury, lead, nickel, and antimony, was based on both biological plausibility and regional relevance. Arsenic, cadmium, and mercury are among the most studied in cardiovascular toxicology, linked to endothelial dysfunction, oxidative injury, and atherosclerotic changes. Lead and nickel have established associations with hypertension and vascular remodeling. Antimony, though less extensively studied, frequently co-occurs with industrial and mining pollutants, serving as a marker of mixed environmental exposure in North India. These metals are prevalent in local groundwater, agricultural soils, and food chains due to industrial effluents and pesticide runoff.

Further evidence for a causal link comes from the Trial to Assess Chelation Therapy (TACT), where EDTA (ethylenediaminetetraacetic acid) chelation known to bind heavy metals significantly reduced cardiovascular events in diabetic patients post-myocardial infarction (MI) [7]. Together, these data underscore the hypothesis that chronic metal exposure contributes to the vascular milieu predisposing one to CAD. Accordingly, this study aimed to evaluate serum levels of the six metals in Indian patients undergoing coronary angiography and to determine their association with angiographic disease complexity and six-month cardiovascular outcomes.

## Materials and methods

Study design

This was a prospective, observational cohort study conducted at a tertiary care hospital (Dr. Ram Manohar Lohia Institute of Medical Sciences, Lucknow) in North India over a period of two years. The primary aim was to assess the association between serum levels of selected heavy metals and angiographic severity of coronary artery disease, while the secondary aim was to evaluate six-month clinical outcomes among participants undergoing diagnostic coronary angiography for suspected or established CAD.

Patient recruitment and eligibility

Patients aged ≥18 years who were scheduled to undergo coronary angiography for either acute coronary syndrome (ACS) or stable CAD were approached for inclusion. Participants had to provide written informed consent for participation and for follow-up over six months. Exclusion criteria included prior coronary revascularization (percutaneous coronary intervention or coronary artery bypass grafting), presence of myocarditis, severe comorbid conditions likely to affect survival within six months (such as advanced malignancy, end-stage renal disease, decompensated liver disease), and angiography revealing normal coronary arteries (no significant obstructive disease). The Institutional Ethics Committee of Dr. Ram Manohar Lohia Institute of Medical Sciences, Lucknow, approved the study protocol.

Data collection

Baseline Clinical and Demographic Data

On enrolment, detailed demographic data (age, sex), traditional cardiovascular risk factors (hypertension, diabetes mellitus, dyslipidemia, family history of CAD, smoking status, alcohol use), lifestyle factors (dietary pattern, physical activity, sleep quality), and clinical presentation (stable angina, acute MI, unstable angina) were recorded using a structured case report form. For hypertension, diagnostic criteria used were a previous diagnosis by a physician or antihypertensive treatment; similarly, diagnosis of diabetes was based on existing medical records or use of hypoglycemic therapy.

Angiography and SYNTAX Scoring

All participants underwent diagnostic coronary angiography per hospital standard protocol. Angiograms were evaluated by interventional cardiologists blinded to the heavy metal measurements. The SYNTAX (Synergy between PCI with Taxus™ and Cardiac Surgery) score was calculated using the standard method (incorporating lesion complexity, location, total occlusions, bifurcation lesions, etc.) [8]. Patients were stratified into two groups: those with a SYNTAX score <23 and those with a score ≥23, to represent lower versus higher angiographic disease complexity.

Heavy Metal Measurement

Fasting blood samples were collected from all participants between 8:00 and 10:00 AM prior to angiography or intervention. The serum was separated and stored at −80 °C until assayed. Concentrations of six heavy metals, arsenic, antimony, mercury, lead, nickel, and cadmium, were quantified using inductively coupled plasma mass spectrometry (ICP-MS). Quality control included the use of certified reference materials, blanks, and repeat measurements for a subset of samples to assess intra- and inter-assay variability.

Outcomes

The primary exposure variables were serum levels of the six heavy metals. The main disease‐severity outcome was the SYNTAX score (dichotomized <23 vs. ≥23). Secondary clinical outcomes were major adverse cardiovascular events (MACE) during six-month follow-up, defined as a composite of all‐cause mortality, non-fatal MI, repeat coronary revascularization, and stroke.

Follow-up and data collection process

Participants were followed up for six months from the date of angiography. Follow-up was done via a combination of in-person clinic visits and telephonic interviews. At each follow-up contact, occurrence of MACE components (death, MI, stroke, repeat revascularization) was ascertained. Medical records were reviewed to confirm events. Lost to follow-up was monitored; attempts were made to contact by phone and home visits where necessary. Data on any changes in medical therapy, interim hospitalizations, or adverse events were also collected.

Sample size calculation

Based on the prior literature indicating a moderate effect size (Cohen’s d ≈0.3) for differences in heavy metal levels between high- and low-SYNTAX score groups, with α = 0.05 (two-tailed) and power = 80%, the minimal sample size required per group was calculated as 176 patients. To allow for subgroup analyses and an anticipated dropout rate of ~20%, a target of at least 480 patients was set. Ultimately, 497 patients were recruited.

Statistical analysis

Statistical analyses were performed using IBM SPSS Statistics, version 27 (IBM Corp., Armonk, USA). Continuous variables are expressed as means ± standard deviations or median (interquartile range) as appropriate. Categorical variables are presented as counts and percentages. Between-group comparisons were made using the independent-samples t-test or Mann-Whitney U test for continuous variables and the χ² test for categorical variables.

Multivariable linear and logistic regression analyses were conducted to evaluate associations between individual metal concentrations and angiographic disease severity or short-term adverse outcomes. The covariates included in all adjusted models were age, sex, hypertension, diabetes mellitus, smoking status, and dyslipidemia, chosen a priori based on established cardiovascular risk relationships.

Missing data were minimal (<5% for all variables) and were handled using listwise deletion. To reduce the likelihood of type I error from multiple comparisons across six metals, Bonferroni correction was applied, setting a two-tailed significance level of p < 0.008 for subgroup comparisons. The remaining analyses were interpreted as exploratory.

## Results

Baseline characteristics

The study had 497 patients with a mean age of 54.58 ± 10.31 years. The study subjects were divided into two study groups: SYNTAX score <23 and SYNTAX score ≥23, with each having 216 and 281 patients, respectively (Table 1).

**Table 1:** Patient demographics and cardiovascular parameters stratified by SYNTAX score BMI: body mass index; SBP: systolic blood pressure; DBP: diastolic blood pressure; HR: heart rate; LVEF: left ventricular ejection fraction; SYNTAX: Synergy between PCI with Taxus™ and Cardiac Surgery (a scoring system to grade coronary artery disease complexity) Boldfaced p-values indicate statistical significance (p < 0.05).

Variable	Mean ± SD (N = 497)	SYNTAX <23 (n = 216), mean ± SD	SYNTAX ≥23 (n = 281), mean ± SD	Mean difference (95% CI)	p-value
SYNTAX score	23.65 ± 11.28	13.94 ± 5.78	31.11 ± 8.43	17.17 (15.51 to 18.83)	-
Age (years)	54.58 ± 10.31	52.69 ± 9.67	56.04 ± 10.58	3.35 (1.55 to 5.15)	<0.001
BMI (kg/m²)	25.62 ± 4.04	26.04 ± 4.28	25.30 ± 3.82	-0.74 (-1.80 to 0.32)	0.304
SBP (mm Hg)	130.3 ± 15.02	129.75 ± 15.25	130.84 ± 14.77	1.09 (-1.82 to 4.00)	0.424
DBP (mm Hg)	76.99 ± 10.09	77.13 ± 9.80	76.90 ± 9.88	-0.23 (-2.91 to 2.45)	0.781
HR (beats/min)	78.11 ± 10.14	78.07 ± 9.88	78.14 ± 10.34	0.07 (-2.01 to 2.15)	0.943
LVEF (%)	49.46 ± 10.69	51.08 ± 10.29	48.21 ± 10.84	-2.87 (-5.00 to -0.74)	0.003

The mean SYNTAX score of the study population was 23.6. The group with a higher SYNTAX score had a higher proportion of patients with higher age, hypertension, and lower ejection fraction. The number of patients with dyslipidemia was higher in the high-SYNTAX score group, though the difference was not statistically significant (p = 0.051). A significant difference was observed between the two groups regarding clinical presentation, with more inferior wall MI patients in the high-SYNTAX score group (Table 2).

**Table 2:** Baseline clinical and lifestyle characteristics by SYNTAX score category CCS/ACS: chronic coronary syndrome/acute coronary syndrome; USA: unstable angina; STEMI/NSTEMI: ST-elevation/non-ST-elevation myocardial infarction; AW STEMI: anterior wall STEMI; IW STEMI: inferior wall STEMI; LW STEMI: lateral wall STEMI; NYHA: New York Heart Association; F/H CAD: family history of coronary artery disease; SYNTAX: Synergy between PCI with Taxus™ and Cardiac Surgery The boldfaced p-value indicates statistical significance (p < 0.05).

Variable		n (%), total N = 497	SYNTAX <23 (n = 216) (%)	SYNTAX ≥23 (n = 281) (%)	OR (95% CI)	p-value
Gender						0.251
Male		396 (79.7)	167 (77.31)	229 (81.4)	1.29 (0.83–2.02)	
Female		101 (20.3)	49 (22.69)	52 (28.6)	0.77 (0.49–1.20)	
Diagnosis						0.002
CCS		142 (28.6)	65 (30.1)	77 (27.4)	0.88 (0.59–1.34)	
ACS		355 (71.4)	151 (69.9)	204 (72.6)	1.14 (0.77–1.69)	
1. USA		20 (4.0)	13 (8.6)	7 (3.43)		
2. NSTEMI		110 (22.1)	52 (34.44)	58 (28.43)		
3. AW STEMI		118 (23.7)	55 (36.42)	63 (30.88)		
4. IW STEMI		81 (16.3)	19 (12.58)	62 (30.39)		
5. LW STEMI		26 (5.2)	12 (7.95)	14 (6.86)		
NYHA class						0.679
I		1 (0.2)	0	1 (3)	–	
II		261 (52.5)	114 (52.53)	147 (52.5)	0.99 (0.69–1.41)	
III/IV		235 (47.3)	102 (47)	133 (47.5)	1.01 (0.71–1.44)	
Symptom duration						0.137
<1 month		183 (36.8)	86 (39.81)	97 (34.52)	0.80 (0.53–1.21)	
1–12 months		202 (40.6)	77 (35.65)	125 (44.48)	1.45 (0.97–2.18)	
>1 year		112 (22.5)	53 (24.54)	59 (21)	0.83 (0.53–1.29)	
Smoker		178 (35.8)	81 (37.5)	97 (34.52)	0.87 (0.58–1.29)	0.492
Diabetics		162 (32.6)	68 (31.48)	94 (33.45)	1.09 (0.74–1.60)	0.835
Hypertension		126 (25.4)	40 (18.52)	86 (30.6)	1.93 (1.25–2.97)	0.005
Dyslipidemic		52 (10.5)	16 (7.41)	36 (12.81)	1.83 (0.98–3.40)	0.051
F/H CAD		129 (26.1)	55 (25.46)	74 (26.33)	1.05 (0.70–1.57)	0.826
Sedentary lifestyle		203 (40.8)	95 (44)	108 (38.43)	0.78 (0.54–1.13)	0.248
Non-vegetarian		263 (52.9)	113(52.31)	150 (53.38)	1.05 (0.73–1.51)	0.813
Alcohol consumption		96 (19.3)	41 (19)	55 (19.57)	1.03 (0.66–1.61)	0.838
Sleep <6 hours		131 (26.4)	60 (27.78)	71 (25.26)	0.88 (0.59–1.31)	0.529
Exercise frequency						0.155
<4 days/week		390 (78.5)	162 (75)	228 (81.14)		
4–6 days/week		106 (21.3)	53 (24.54)	53 (18.86)	0.72 (0.48–1.09)	
Daily		1 (0.2)	1 (0.46)	0		

Other baseline characteristics, such as duration of symptoms, number of smokers/alcoholics/diabetics, family history of CAD, sedentary lifestyle, diet pattern, sleep dysfunction, and exercise, were evenly distributed among the two groups without any statistical difference (Table 2).

Heavy metal levels and SYNTAX score

There was no statistically significant difference in the mean serum levels of arsenic, antimony, mercury, lead, nickel, or cadmium between the two SYNTAX groups. These findings suggest that the overall angiographic severity of CAD may not directly correlate with blood heavy metal concentrations (Table 3).

**Table 3:** Mean heavy metal levels and angiographic severity of CAD SYNTAX: Synergy between PCI with Taxus™ and Cardiac Surgery; CAD: coronary artery disease; NS: not significant

Heavy metal		Overall (N = 497)	SYNTAX <23 (mean ± SD)	SYNTAX ≥23 (mean ± SD)	Mean difference (95% CI)	p-value
Arsenic (µg/L)		4.40 ± 6.02	4.38 ± 6.26	4.44 ± 6.26	0.06 (-1.11 to 1.23)	NS
Antimony (µg/L)		1.58 ± 3.21	1.47 ± 2.88	1.67 ± 3.44	0.20 (-0.37 to 0.77)	NS
Mercury (µg/L)		13.06 ± 17.54	14.04 ± 19.96	12.30 ± 15.42	-1.74 (-5.06 to 1.58)	NS
Lead (µg/dL)		2.18 ± 4.13	2.10 ± 3.69	2.23 ± 4.45	0.13 (-0.64 to 0.90)	NS
Nickel (µg/L)		7.95 ± 19.20	8.10 ± 17.24	7.83 ± 20.60	-0.27 (-3.50 to 2.96)	NS
Cadmium (µg/L)		3.90 ± 4.55	3.85 ± 4.54	3.94 ± 4.57	0.09 (-0.53 to 0.71)	NS

Subgroup Analysis

When subgroup analysis was performed by grouping the patients as per their clinical presentation, patients with ACS had significantly higher serum cadmium levels compared to those with stable CAD (p = 0.006). The difference was limited to cadmium as other heavy metals under study did not differ between the ACS and the stable CAD groups to achieve significance (Table 4).

**Table 4:** Heavy metal levels in stable CAD versus ACS patients CAD: coronary artery disease; ACS: acute coronary syndrome The boldfaced p-value indicates statistical significance (p < 0.05).

Heavy metal	Stable CAD (n = 142), mean ± SD	ACS (n = 355), mean ± SD	Mean difference (95% CI)	p-value
Arsenic (µg/L)	4.34 ± 6.09	4.48 ± 6.04	0.14 (-1.45 to 1.73)	0.81
Antimony (µg/L)	1.58 ± 2.91	1.58 ± 3.36	0.00 (-0.74 to 0.74)	0.99
Mercury (µg/L)	11.37 ± 17.63	13.87 ± 17.48	2.50 (-1.03 to 6.03)	0.13
Lead (µg/L)	1.84 ± 3.67	2.34 ± 4.33	0.50 (-0.42 to 1.42)	0.21
Nickel (µg/L)	7.05 ± 20.22	8.36 ± 18.67	1.31 (-3.26 to 5.88)	0.47
Cadmium (µg/L)	3.06 ± 3.78	4.35 ± 4.78	1.29 (0.38 to 2.20)	0.006

Heavy metal and its association with risk factors

Hypertensive patients demonstrated significantly elevated levels of arsenic (p = 0.001) and mercury (p = 0.0013) when compared to normotensive individuals, suggesting a potential link between chronic heavy metal exposure and hypertensive vascular damage. No significant associations were observed between heavy metal levels and other risk factors such as smoking, alcohol use, diabetes, or dyslipidemia, except for antimony, which was marginally higher in smokers (p = 0.04) (Table 5).

**Table 5:** Heavy metal concentrations by cardiovascular risk factors Boldfaced p-values indicate statistical significance (p < 0.05).

Risk factor		Arsenic (µg/L), mean ± SD	Antimony (µg/L), mean ± SD	Mercury (µg/L), mean ± SD	Lead (µg/L), mean ± SD	Nickel (µg/L), mean ± SD	Cadmium (µg/L), mean ± SD
Smoking	No (n = 319)	4.66 ± 6.31	1.36 ± 2.82	12.99 ± 11.72	7.67 ± 17.88	8.41 ± 21.12	3.92 ± 4.52
	Yes (n = 178)	4.04 ± 5.53	1.97 ± 3.79	13.15 ± 18.53	8.92 ± 16.14	7.07 ± 15.14	3.96 ± 4.53
	p-value	0.27	0.04	0.92	0.57	0.45	0.92
	Diabetes mellitus	No (n = 332)	4.19 ± 5.74	1.74 ± 3.54	13.39 ± 19.05	7.67 ± 17.95	7.64 ± 17.30	3.71 ± 4.41
Yes (n = 165)		4.91 ± 6.66	1.26 ± 2.42	12.30 ± 14.17	8.06 ± 23.18	7.77 ± 20.30	4.42 ± 4.74
p-value		0.22	0.12	0.52	0.81	0.94	0.09
Hypertension		No (n = 370)	3.62 ± 5.37	1.66 ± 3.19	11.58 ± 17.49	7.64 ± 18.11	8.39 ± 20.67	3.81 ± 4.61
	yes (n = 127)	6.79 ± 7.23	1.35 ± 3.29	17.36 ± 17.12	8.12 ± 23.32	6.61 ± 13.98	4.31 ± 4.25
	p-value	0.001	0.35	0.001	0.86	0.37	0.29

Six-month outcomes and MACE comparison between low- and high-SYNTAX score groups

At the six-month follow-up, MACE were significantly more frequent in patients with higher angiographic disease burden (SYNTAX score ≥23). Out of 497 patients, 33 experienced a major adverse cardiovascular event (6.64%) (Table 6).

**Table 6:** Clinical outcomes by SYNTAX score category MI: myocardial infarction; CVA: cerebrovascular accident (stroke); SYNTAX: Synergy between PCI with Taxus™ and Cardiac Surgery

Outcome	SYNTAX <23, no. of events (%)	SYNTAX ≥23, no. of events (%)	OR (95% CI)	p-value
Death	2 (0.9)	15 (5.3)	6.14 (1.32–28.5)	0.007
MI	1 (0.5)	7 (2.5)	5.12 (0.59–44.3)	0.075
Revascularization	1 (0.5)	5 (1.8)	3.61 (0.39–33.3)	0.183
CVA	1 (0.5)	1 (0.4)	0.77 (0.05–12.1)	0.852
Total	5 (2.3)	28 (10.0)	4.70 (1.72–12.9)	<0.01

**Figure 1:**
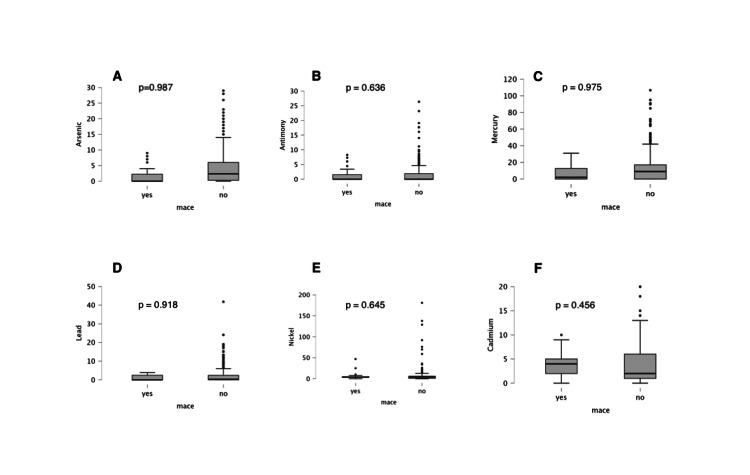
Heavy metal levels in patients with and without major adverse cardiovascular events (MACE): (A) arsenic), (B) antimony, (C) mercury, (D) lead, (E) nickel, and (F) cadmium No significant difference could be seen, as suggested by p > 0.05.

Patients with a SYNTAX score ≥23 had significantly higher all-cause mortality (5.3% vs. 0.9%, p = 0.007) and total MACE (10.0% vs. 2.3%). While MI and repeat revascularization were more frequent in the high-SYNTAX score group, the differences did not reach statistical significance (p = 0.075 and 0.183, respectively). Stroke incidence was low and similar in both groups (p = 0.852). However, there was no significant difference in MACE in patients with regard to serum levels of heavy metals (Figure 1).

## Discussion

This study is one of the largest Indian cohort studies to examine the relationship between heavy metal exposure and CAD severity. In this prospective observational study of 497 patients undergoing coronary angiography for suspected CAD, we assessed the association between serum concentrations of selected heavy metals and angiographic complexity using the SYNTAX score.

In this study, serum concentrations of six heavy metals, arsenic, antimony, cadmium, mercury, lead, and nickel, showed no significant association with anatomical disease complexity as quantified by the SYNTAX score. However, important subgroup associations emerged. Cadmium levels were significantly higher in patients presenting with ACS compared to those with stable CAD. Arsenic and mercury concentrations were elevated in patients with hypertension. These findings suggest that while heavy metal burden may not directly influence total atherosclerotic plaque volume, it could modulate plaque stability, vascular reactivity, and systemic hemodynamic patterns.

Heavy metals such as cadmium, mercury, and arsenic are known to increase oxidative stress, impair endothelial function, disrupt nitric oxide signaling, and elevate inflammatory markers. These effects may not necessarily manifest as an extensive atherosclerotic burden, as captured by the SYNTAX score, but may instead promote plaque instability, microvascular dysfunction, and vascular stiffness. This could explain why cadmium was linked with ACS and arsenic/mercury with hypertension, without showing a direct association with anatomical disease complexity.

The adverse cardiovascular effects of heavy metals such as cadmium, arsenic, and mercury are underpinned by a well-characterized constellation of cellular and molecular pathways. These metals disrupt redox homeostasis, primarily by promoting the generation of ROS. The resulting oxidative stress leads to impaired eNOS activity, thus causing reduced nitric oxide bioavailability, which is a critical mediator of vascular tone and endothelial health.

Cadmium is known to accumulate in vascular tissue, where it inhibits antioxidant enzymes like superoxide dismutase (SOD) and glutathione peroxidase. It also promotes lipid peroxidation and disrupts calcium signaling in vascular smooth muscle cells, which can increase vasoreactivity and potentially trigger vasospasm or plaque rupture, mechanisms that may explain its association with acute coronary syndrome in our cohort.

Arsenic exerts its vascular toxicity by activating pro-inflammatory transcription factors such as NF-κB and AP-1, upregulating adhesion molecules (VCAM-1, ICAM-1), and inducing endothelial apoptosis [9]. Chronic low-level exposure has been shown to increase vascular stiffness and promote a prothrombotic state, in part through impaired endothelial repair mechanisms.

Mercury, particularly in its methylated form, can cross the blood-brain and endothelial barriers, disrupt mitochondrial function, and amplify systemic oxidative and inflammatory responses [10]. It has been linked to increased sympathetic activity, hypertension, and carotid intima-media thickening in experimental and human studies [11]. The elevated levels of mercury in hypertensive patients in our study are biologically plausible in this context.

Notably, these metals may not uniformly contribute to atherosclerotic plaque volume but rather influence plaque composition and stability, vascular reactivity, and microvascular dysfunction [12-13]. This nuanced mechanism could explain why no direct correlation was observed with angiographic SYNTAX scores, but clinically relevant associations emerged in ACS and hypertensive subgroups.

Our patient cohort had a mean age of 54.58 years, younger than the populations described in studies from Iran and Europe, where the average patient age often exceeded 65 years [14-15]. This aligns with earlier Indian studies by Joshi et al. and Nair and Prabhakaran, where premature CAD among South Asians was noted [3-4]. The proportion of hypertensives (~25%) and diabetics (~33%) in our study was comparable to other Indian cohorts [16]. This younger demographic reflects the well-established trend of premature CAD in the Indian population. While Western cohorts, with mean ages over 65, represented chronic stable atherosclerosis, our study captured clinically unstable CAD in a younger group, highlighting the importance of exploring non-traditional risk factors like environmental exposures in such populations.

With 56.5% of patients having a SYNTAX score ≥23, our cohort shows among the highest reported anatomical CAD burden in India, especially for a younger population (mean age ~54.58). This proportion is similar to what has been reported in comparable Indian studies. A study by Satheesh et al. reported high SYNTAX scores in 64.4% patients of the 1000 patients [16]. The high complexity in our younger cohort suggests a more aggressive disease phenotype, potentially influenced by genetic, metabolic, and environmental factors.

Despite high disease complexity, we found no significant differences in mean serum levels of arsenic, antimony, cadmium, mercury, lead, and nickel across SYNTAX groups. However, subgroup analysis revealed key associations: patients presenting with acute coronary syndrome had significantly higher serum cadmium levels (p = 0.006), while hypertensive patients had significantly elevated levels of arsenic (p = 0.001) and mercury (p = 0.0013). These findings support the concept that although serum levels of heavy metals do not directly correlate with angiographic severity of CAD, they may influence disease stability, plaque vulnerability, and clinical presentation.

Our results align with findings of Sponder et al., who reported no relationship between blood heavy metal levels and CAD severity in stable CAD, but contrast with those of Asgary et al., who observed higher cadmium, mercury, and lead levels in Iranian CAD patients [14-15]. The National Health and Nutrition Examination Survey (NHANES) dataset and the TACT trial support a broader link between heavy metal burden and cardiovascular events, particularly in high-risk subgroups [17-18]. The present study extends this evidence to a large, relatively young Indian cohort, showing that cadmium, arsenic, and mercury may influence clinical phenotypes (ACS, hypertension) rather than purely anatomical severity.

Our finding that cadmium was associated with ACS aligns with experimental studies showing its potential to trigger plaque rupture. The systematic review by Nucera et al. also found cadmium to be a major factor leading to increased ischemic events [19]. Similarly, the higher arsenic and mercury levels in hypertensives correspond to their known vascular effects, namely, impairment of nitric oxide synthesis, vascular stiffness, and sympathetic overactivation. A systematic review reported that greater arsenic exposure is associated with a higher prevalence of hypertension, supported by dose-response evidence in multiple populations, including Bangladesh and Taiwan [20]. A narrative review by Hu et al. highlighted a strong dose-response relationship, especially at hair Hg levels above ~2 μg/g, and clear biological plausibility (oxidative stress, endothelial dysfunction, and renin-angiotensin-aldosterone system, or RAAS, modulation) [21].

Our six-month outcome data revealed significantly higher mortality (p = 0.007) and total MACE (p < 0.01) in patients with a SYNTAX score ≥23. While individual events such as MI and revascularization were more frequent in this group, they did not reach statistical significance, likely due to limited sample size. These findings align with large registries demonstrating that elevated SYNTAX scores are predictive of adverse outcomes. For instance, Safarian et al. reported a MACE rate of 21.6% in the highest SYNTAX tertile compared to 7.5% in the lowest (hazard ratio ≈2.36, p = 0.02) [22]. Similarly, Capodanno et al. found high anatomical complexity to be independently associated with worse prognosis in ACS patients [23]. Xu et al. further confirmed the long-term prognostic value of SYNTAX scores in unstable angina populations [24]. Together, these studies validate the clinical utility of SYNTAX scoring in risk stratification.

Compared to prior Indian studies [25], our MACE rate in the SYNTAX score ≥23 patients (10%) was consistent with that reported in high-risk diabetic populations and far exceeded that in low-SYNTAX score groups. This adds weight to the need for aggressive risk factor control and perhaps early revascularization in patients with more severe CAD.

To conclude, while overall heavy metal levels did not correlate with the SYNTAX score, exploratory subgroup analyses revealed that the cadmium level was higher in ACS patients and arsenic/mercury levels were elevated in hypertensive participants. These observations are hypothesis-generating and require confirmation in larger, multicenter cohorts before causal inference can be made.

Clinical implications

Our findings highlight that heavy metal exposure, particularly to cadmium, arsenic, and mercury, may influence the clinical presentation of coronary artery disease, such as acute coronary syndrome and hypertension, even in the absence of increased anatomical disease complexity. This suggests that traditional angiographic scores like SYNTAX may not fully capture the vascular impact of environmental toxins.

Given the high burden of premature CAD and widespread unregulated exposure in India, incorporating environmental risk assessment, especially in younger patients or those with unexplained hypertension or ACS, could enhance cardiovascular risk stratification. Furthermore, public health strategies aimed at reducing toxic metal exposure through improved environmental monitoring, regulation, and dietary safety may have a role in primary prevention.

Strengths and limitations

The strengths of this study include its relatively large sample size and prospective design, which together allowed for more reliable estimates of the relationship between serum heavy metal burden and CAD severity and short-term outcomes. The use of the SYNTAX score provided an objective, anatomically detailed measure of disease complexity derived from coronary angiography. Measurement of a broad panel of heavy metals using ICP-MS with quality control procedures enhanced the validity of exposure quantification. Moreover, following up patients for six months allowed us to capture clinically meaningful cardiovascular events in the near term, linking exposures with outcomes beyond mere anatomical observations.

Nonetheless, several limitations exist. Being a single-center study, the findings may have limited generalizability to other geographic regions or populations with different environmental exposures. Since heavy metal levels were measured only at one time point, temporal variation or cumulative exposure could not be assessed. The lack of direct assessment of environmental or dietary sources of metal exposure prevented understanding of exposure pathways. Furthermore, the follow-up period was relatively short, which might have underpowered the detection of longer term MACE. Finally, while adjustments were made for major conventional risk factors, residual confounding by unmeasured factors (e.g., renal function, environmental pollution indices, socio-economic status) cannot be excluded.

The study relied on a single blood draw to estimate heavy metal exposure, which may not fully reflect cumulative lifetime burden. However, prior studies indicate that circulating levels serve as valid biomarkers of biologically active exposure influencing endothelial function and oxidative stress. We interpret our findings as reflecting the current metabolic exposure window rather than total body stores. Prospective studies incorporating repeated measures, urinary excretion data, or hair/nail biomonitoring could provide more robust estimates of cumulative exposure.

Future directions

Our findings open several meaningful avenues for future research. Larger, multicenter studies with diverse populations and extended follow-up could help clarify the clinical impact of heavy metal exposure. Incorporating long-term exposure markers and environmental assessments may offer deeper insights. Exploring whether reducing toxic metal burden can improve cardiovascular outcomes, especially in younger, high-risk individuals, remains an important next step.

## Conclusions

In our study, we did not find a direct link between heavy metal levels and SYNTAX score. However, this does not preclude the role of heavy metals in disease progression. While overall heavy metal levels did not correlate with the SYNTAX score, we observed higher cadmium levels in ACS patients and elevated arsenic and mercury levels in hypertensive patients. These findings suggest that while heavy metals may not influence the extent of coronary blockages, they could still play a role in plaque instability or vascular dysfunction.

Given the rising burden of heart disease in India and the widespread but often overlooked exposure to environmental toxins, this area deserves more attention. Larger, multicentric studies with longer follow-up and mechanistic evaluations are needed to understand how these metals contribute to cardiovascular risk and outcomes. For now, our results support the idea that environmental exposures may silently influence how heart disease presents and progresses in high-risk populations.
